# The Role of Sink Strength and Nitrogen Availability in the Down-Regulation of Photosynthetic Capacity in Field-Grown *Nicotiana tabacum* L. at Elevated CO_2_ Concentration

**DOI:** 10.3389/fpls.2017.00998

**Published:** 2017-06-09

**Authors:** Ursula M. Ruiz-Vera, Amanda P. De Souza, Stephen P. Long, Donald R. Ort

**Affiliations:** ^1^Carl R. Woese Institute for Genomic Biology, University of Illinois at Urbana-ChampaignUrbana, IL, United States; ^2^Department of Plant Biology, University of Illinois at Urbana-ChampaignUrbana, IL, United States; ^3^Lancaster Environment Centre, Lancaster UniversityLancaster, United Kingdom; ^4^Global Change and Photosynthesis Research Unit, Agricultural Research Service, United States Department of AgricultureUrbana, IL, United States

**Keywords:** photosynthetic acclimation, climate change, rising CO_2_, C_3_ photosynthesis, Rubisco, Free Air CO_2_ Enrichment, nitrogen fertilization, sink limitation

## Abstract

Down-regulation of photosynthesis is among the most common responses observed in C_3_ plants grown under elevated atmospheric CO_2_ concentration ([CO_2_]). Down-regulation is often attributed to an insufficient capacity of sink organs to use or store the increased carbohydrate production that results from the stimulation of photosynthesis by elevated [CO_2_]. Down-regulation can be accentuated by inadequate nitrogen (N) supply, which may limit sink development. While there is strong evidence for down-regulation of photosynthesis at elevated [CO_2_] in enclosure studies most often involving potted plants, there is little evidence for this when [CO_2_] is elevated fully under open-air field treatment conditions. To assess the importance of sink strength on the down-regulation of photosynthesis and on the potential of N to mitigate this down-regulation under agriculturally relevant field conditions, two tobacco cultivars (*Nicotiana tabacum* L. cv. Petit Havana; cv. Mammoth) of strongly contrasting ability to produce the major sink of this crop, leaves, were grown under ambient and elevated [CO_2_] and with two different N additions in a free air [CO_2_] (FACE) facility. Photosynthetic down-regulation at elevated [CO_2_] reached only 9% in cv. Mammoth late in the season likely reflecting sustained sink strength of the rapidly growing plant whereas down-regulation in cv. Petit Havana reached 25%. Increased N supply partially mitigated down-regulation of photosynthesis in cv. Petit Havana and this mitigation was dependent on plant developmental stage. Overall, these field results were consistent with the hypothesis that sustained sink strength, that is the ability to utilize photosynthate, and adequate N supply will allow C_3_ crops in the field to maintain enhanced photosynthesis and therefore productivity as [CO_2_] continues to rise.

## Introduction

Due to anthropogenic activities, the CO_2_ concentration ([CO_2_]) of the atmosphere has risen dramatically since 1750 (IPCC, 2013); currently increasing at annual rate average of 2.1 μmol mol^−1^ (NOAA, 2016). For C_3_ plants, rising [CO_2_] increases the potential net leaf rate of photosynthetic CO_2_ uptake (*A*) since the current [CO_2_] of 400 μmol mol^−1^ is insufficient to saturate ribulose-1,5-bisphosphate carboxylase/oxygenase (Rubisco), and because rising [CO_2_] competitively depresses the oxygenase activity of Rubisco and, in turn, photorespiration (Long, 1991; Drake et al., 1997). In Free Air CO_2_ Enrichment (FACE) experiments, elevating the ambient atmospheric [CO_2_] by 100–250 μmol mol^−1^ resulted in an increase in *A* of 13–46% depending on the level of [CO_2_] elevation, plant functional group, and interacting environmental factors (Ainsworth and Long, 2005; Leakey et al., 2009). This increased potential is seldom fully realized across the growing season due to down-regulation of photosynthesis capacity that occurs when C_3_ plants are grown at elevated [CO_2_] (Drake et al., 1997; Moore et al., 1999; Rogers and Humphries, 2000; Ainsworth and Long, 2005; Bernacchi et al., 2005). Down-regulation of photosynthetic capacity at elevated [CO_2_] is routinely attributed to insufficient sink capacity (i.e., storage and other heterotrophic tissues) to utilize the additional photosynthate (Drake et al., 1997; Ainsworth et al., 2004) produced as a consequence of the increased [CO_2_].

At elevated [CO_2_], carbohydrates can accumulate in source leaves and signal the repression of genes for photosynthetic proteins such as *rbcS* leading to down-regulation of *A* (Drake et al., 1997; Moore et al., 1999). Since photosynthetic proteins, like Rubisco, account for much of N in crop leaves, it is unsurprising that down-regulation in elevated [CO_2_] is frequently accompanied by reduction in leaf nitrogen (N) content (Rogers et al., 1996; Drake et al., 1997; Moore et al., 1999; Long et al., 2004; Leakey et al., 2009). It has also been frequently observed that down-regulation of photosynthesis at elevated [CO_2_] is greater when N is limiting (Petterson and McDonald, 1994), since N-deficiency will limit growth and activity of sink tissues. Nitrogen may also have a feed-forward influence on source:sink balance as less N in the leaves results in less carbohydrate incorporated into amino acids, which in turn may reduce the capacity for sugar transport from sources to sinks (Paul and Driscoll, 1997). Thus, preventing an escalation in the carbon:nitrogen (C:N) ratio may be critical to maintain an equilibrium between production and utilization of carbohydrates and to avoid a state of sink limitation that potentiates down-regulation in photosynthesis (Paul and Driscoll, 1997; Leakey et al., 2009).

The effects of the sink-source relationship in photosynthesis for plants grown under elevated [CO_2_] conditions has been previously studied, most of the time in enclosed environments such as greenhouses, growth chambers and open-top chambers and often with potted plants (e.g., von Caemmerer and Farquhar, 1984; Arp, 1991; McConnaughay et al., 1993; Farage et al., 1998; Ainsworth and Rogers, 2007; Burnett et al., 2016). The use of pots can restrict the growth of sinks organs, like roots (Arp, 1991), and thus be a poor surrogate for understanding impacts of elevated [CO_2_] on field crops. Results from enclosures, perhaps through modified micrometeorological conditions, have different crop responses to elevated [CO_2_] than those observed under fully open air conditions (Ainsworth et al., 2008). Few studies have directly investigated the sink limitation hypothesis for photosynthetic down-regulation in field FACE experiments, which avoid the uncertainties from both pot restrictions and enclosure environments (e.g., Bryant et al., 1998; Rogers et al., 1998, 2004; Ainsworth et al., 2003, 2004). With the continuous increase of atmospheric [CO_2_], it is critical to understand the role of sink limitation in the down-regulation of photosynthetic capacity under agricultural field conditions and the capacity of N availability to mitigate it if agriculture is to meet future demand (Long et al., 2004; Tilman and Clark, 2015). This raises the questions of whether by genetically increasing sink size and providing sufficient N, can down-regulation be avoided and the full potential photosynthetic benefit of rising [CO_2_] be realized in crops?

In this study, the importance of sink strength on photosynthetic down-regulation and the potential of N to mitigate down-regulation was assessed in tobacco (*Nicotiana tabacum* L.) using replicated Free Air CO_2_ Enrichment (FACE) treatments. Because the major sink for assimilate in cultivated tobacco is the leaf, we used two contrasting genotypes at the extremes of sink capacity: cv. Petit Havana, which as the name suggests produces relatively small leaves and flowers early under Illinois conditions; and cv. Mammoth, which as the name suggests produced very large leaves and does not flower until late fall, so continues producing new leaves throughout the entire summer growing season. The experiment was conducted with two different N additions. We hypothesized that with elevated [CO_2_] cv. Mammoth with nitrogen addition would show little or no down-regulation of photosynthesis compared to cv. Petit Havana and that the down-regulation would be greater in both cultivars under limiting N conditions. Leaf gas exchange data combined with carbohydrate analysis, leaf carbon and nitrogen content, growth measurements and yield data were collected and analyzed to determine the factors that could drive the down-regulation of apparent Rubisco activity *in vivo* (*V*_*cmax*_) in tobacco. We found that down-regulation of photosynthesis at elevated [CO_2_] was least in cv. Mammoth consistent with this cultivar's sustained sink strength of this rapidly growing indeterminant genotype. However, down-regulation in cv. Petit Havana was so strong, that on the last measurement date *A* in plants grown and measured under elevated [CO_2_] was less than that in the plants grown and measured in ambient [CO_2_]. Increased N partially mitigated down-regulation of photosynthesis in cv. Petit Havana nevertheless *A* was still lower in the plants grown and measured at elevated [CO_2_].

## Materials and methods

### Field site, tobacco cultivars, and experimental design

The experiment was performed in the summer of 2015 at the Soybean Free-Air CO_2_ Enrichment (SoyFACE) facility, in Champaign, IL, USA (40°2′30.5″N, 88°13′58.8″W, 230 m a.s.l.). Two tobacco cultivars *N. tabacum* L. cv. Petit Havana (PH) and cv. Mammoth (MM) were used. PH seeds were obtained from the Australian National University while MM seeds with variety name “Kutsaga Mammoth 10” were obtained from the US Nicotiana Germplasm Collection, North Carolina State University. Tobacco seeds were sown in seedling trays (200 cell Speedling Transplant Trays, Speedling, FL, USA) filled with a germination mix soil (Fafard, MD, USA). Germination occurred 7 days after the sowing, on the day of the year (DOY) 173 (Table S1). The environmental conditions in the greenhouse were: natural sunlight, 80% relative humidity (RH), and an average temperature of 26°C during the day and 24°C at night. The seedlings received NPK fertilization (20-10-20 Jack's General Purpose Professional, JR PETERS Inc., PA, USA) and applications of the broad spectrum systemic fungicides (Dithane, Dow Agrochemicals and Terramaster 4EC, Chemtura). After 4 weeks, the seedlings were ~5 cm tall, at which stage they were transplanted into the field.

The field contained four 20 m diameter FACE treatment areas in which [CO_2_] was elevated to 600 μmol mol^−1^ (Morgan et al., 2004). Each of these was paired with a second 20 m diameter area, which served at the ambient [CO_2_] (400 μmol mol^−1^) control of the experimental block. Within each of these areas a plot of 32 m^2^ was allocated to the current experiment (Figure S1). Each plot was split with one half planted with cv. Petit Havana (PH) and the other half with cv. Mammoth (MM). These areas were in turn split into a control (CN) and high nitrogen treatment (HN), giving a split-split-plot design of N × cultivar × [CO_2_]. The treatments are denoted as follows: ambient [CO_2_] with control nitrogen (AMB CN); ambient [CO_2_] with high nitrogen (AMB HN); elevated [CO_2_] with control nitrogen (ELE CN); elevated [CO_2_] with high nitrogen (ELE HN).

The soil in the field was cultivated to a fine tilth a week before transplantation. The split plots (subplots) were planted at a row spacing of 30.5 cm for PH (6.56 plants/m^2^) and 38.1 cm for MM (5.25 plants/m^2^), to allow for the larger size of the latter. Insecticide (Platinum 75SG, Syngenta Crop Protection Inc., NC, USA) was applied on the planting day (DOY 195, Table S1). On the day before field planting (DOY 194, Table S1), the soil inside the experimental plots was fertilized. One half of each cultivar received a standard fertilization with 150 Kg N/ha (CN) and the other 300 kg N/ha (HN) in the form of urea (UREA 46-0-0, J.R. Simplot Company, CA, USA; Figure S1). The N content of the soil before fertilization was on average 17.2 ± 3.4 ppm of NO_3_ and 3.0 ± 0.6 ppm of NH_4_. Four days after the addition of fertilizer, the soils ontained 55.2 ± 9.9 ppm NO_3_ with 36.5 ± 13.4 ppm NH_4_ (CN) and 81.8 ± 11.6 ppm of NO_3_ with 83.57 ± 28.6 ppm of NH_4_ (HN). Soil moisture was maintained during the experiment through drip irrigation to maintain soil volumetric water content between 32 and 40% _v/v_ throughout the season.

### Gas exchange measurements

Leaf CO_2_ uptake rates (*A*, μmol CO_2_ m^−2^ s^−1^), stomatal conductances (*g*_*s*_, mol H_2_O m^−2^ s^−1^) and ratio of internal [CO_2_] (*C*_*i*_) to ambient [CO_2_] (*C*_*a*_) were determined on four occasions separated by 1 or 2 week intervals across the growing season (Table S1). On each of these days, measurements were made at 09:00, 12:00, and 15:00 h using open gas-exchange systems incorporating a controlled environment leaf chamber that integrated a modulated chlorophyll fluorometer (LI-6400 & LI-6400-40; LICOR, Inc., Lincoln, NE, USA). Before each time-point, the values for the photosynthetically active radiation (PAR; μmol m^−2^s^−1^) and chamber block temperature were set at the ambient values determined from the SoyFACE weather station (sensors: LI-190; LI-COR, Inc. and HMP-45C; Campbell Scientific, Inc.). Daily air temperature and PAR across the growing season are presented in Figure S2. The reference [CO_2_] in the gas-exchange systems was set to 400 μmol mol^−1^ for the AMB treatments and to 600 μmol mol^−1^ for the ELE treatments. At each time-point, one of four gas-exchange systems was assigned at random to each of the four blocks, to avoid confounding effects of any undetected difference among gas exchange systems with any difference among blocks. The youngest fully expanded leaves from three plants per subplot were measured. The daily mean for relative humidity inside the leaf chamber was between 57% and 69% for both cultivars. The gas exchange systems were calibrated as described previously (Bernacchi et al., 2006), and the calculation of *A, g*_*s*_, and *C*_*i*_/C_*a*_ followed the procedures of von Caemmerer and Farquhar (1981).

*A* vs. *C*_*i*_ response was measured and analyzed following the procedures of Long and Bernacchi (2003). These measurements were also made on the youngest fully expanded leaves in two plants per subplot. This was done three times during the season, at DOY 212, 227, and 240 (Table S1). Because of rain and the difficulty of maintaining constant conditions in the field, the measurements on DOY 227 and DOY 240 were conducted in the field laboratory within the site, rather than *in situ*. For the field laboratory measurements of the *A/C*_*i*_ curves, leaves were cut predawn and the petioles were recut under water to avoid embolism. All leaves were kept under low light until measured in the laboratory. On DOY 220 these measurements were made *in situ* in the field and then repeated in the laboratory the next day, and found not to differ (Figure S3). This procedure also avoids the risk of photoinhibition and transient water stress, that can occur under field conditions. All measurements were completed within 8 h of cutting and maximum rates at 8h still equaled or exceeded those measured in the field. For *A/C*_*i*_ measurements, the PAR level was set at 1500 μmol mol^−1^, leaf temperature at 25°C and relative humidity was set between 60 and 70%. Photosynthesis was induced to steady state in the ambient [CO_2_] of 400 μmol mol^−1^. The chamber [CO_2_] was varied through the following step-wise sequence and waiting for steady-state *A* to be achieved at each step: 400, 300, 200, 100, 400, 400, 500, 600, 800, 1,000, 1,200, and 1,500 μmol mol^−1^. The maximum rate of Rubisco-catalyzed carboxylation (*V*_*cmax*_; μmol m^−2^s^−1^), the regeneration of ribulose-1,5-biphosphate controlled by electron transport rate (*J*_*max*_; μmol m^−2^s^−1^) were calculated from the *A/C*_*i*_ response for each individual using the equations from Farquhar et al. (1980) and Bernacchi et al. (2001). Estimates of *V*_*cmax*_ from *A*/C_*i*_ curves are in effect “apparent *V*_*cmax*_” since they do not take account of mesophyll conductance (*g*_*m*_).

The *g*_*m*_ (mol m^2^ s^−1^) and partial preasure of CO_2_ inside the chloroplast (*C*_*c*_; μmol mol^−1^) were calculate using variable J method as described in Harley et al. (1992) for DOY 240, the only day where chlorophyll fluorescence data was colletect. *V*_*cmax*_, *J*_*max*_, and day respiration independent of photorespiration (*R*_*d*_) were calculated from *A*/C_*c*_ curves as in Harley et al. (1992) and by using a nonlinear analysis with the Marquardt method (PROC NLIN, SAS System 9.4, SAS Institute, Cary, NC, USA; Moualeu-Ngangue et al., 2017). The scaling constant (*c*) and enthalpies of activation (Δ*H*_*a*_) for fitting the Michaelis constant of Rubisco for carbon dioxide (*K*_*c*_; μmol mol^−1^), inhibition constant (*K*_*o*_; μmol mol^−1^), and photorespiratory compensation point (T^*^; μmol mol^−1^) at the measured leaf temperature as well as for normalizing *V*_*cmax*_ and *J*_*max*_ at 25°C were obtained from Sharkey et al. (2007).

### Height, specific leaf area and leaf carbon and nitrogen content

Measurements for plant height (cm) were taken at approximately 1 week intervals, from the soil to the tip of the newest leaf in three plants in each subplot. The specific leaf area (SLA, m^2^ kg^−1^) was calculated from three plants per subplot. Leaf disks of 1.9 cm of diameter were collected at midday on each day of the diurnal measurements of leaf gas exchange measurements. The samples were oven dried to constant weight at 60°C. These leaf disks were subsequently used for CN analysis. The tissue was ground to a powder and a ~2.0 mg sub-sample processed through an elemental analyzer (Elemental Combustion System CHNS-O, Costech ECS 4010, Valencia, CA, USA).

### Leaf carbohydrates

Leaf disk samples (~1.2 cm diameter) were collected from two plants per subplot at noon and dusk on the same days as the diurnal leaf gas exchange measurements. Dawn samples were also collected one of these days, DOY 227 (Table S1). The samples were cut into liquid N in the field and stored at −80°C until analysis. Soluble carbohydrates determination followed the procedures of Ainsworth et al. (2007), excepting the use of an additional ethanol wash. Glucose, fructose, sucrose and starch were expressed as glucose equivalents. Total soluble carbohydrates (TSC) were calculated as the sum of glucose, fructose, and sucrose.

### Plant dry weight

Two harvests were conducted to determine productivity; one in the middle of the season (starting on DOY 222, ~3.5 weeks after field planting), and one just before the PH pods opened (starting on DOY 243, 7 weeks after field planting, Table S1). The above-ground biomass of five plants per subplot was collected in each harvest. The number of leaves, branches and the total leaf area (total LA in cm^2^; measured using an LI-3100, LICOR, Inc.) for each plant were also determined. The roots of the harvested plants were collected a day after the above-ground biomass harvest. The roots of 3 plants per subplot were taken in the 1st harvest and of 2 plants per subplot in the 2nd harvest. Soil was washed from the roots, and these together with leaves, stems, and floral structures then dried to constant weight at 60°C.

### Statistical analysis

All the variables were analyzed as a split-split-plot design using a mixed model analysis of variance (ANOVA; PROC MIXED, SAS System 9.4, SAS Institute, Cary, NC, USA) with repeated measurements, except for the daily analysis of *V*_*cmax*_ and *J*_*max*_, which were analyzed without repeated measurements. The fixed effects were time, [CO_2_], nitrogen (N), and their interactions. Time was the repeated measurement, and it refers to DOY for the seasonal analysis and to the time-points (3 in each day of measurements) for the daily analysis of the leaf gas exchange variables (*A, g*_*s*_, and *C*_*i*_*/C*_*a*_). The random effect for all the variables was the block. The calculation of the degrees of freedom was done using the Kenward–Roger method. The least square means test (*t*-test) was used to obtain the pair-wise comparisons, considering the statistical significance as *P*-value ≤ 0.1. Because of the low replication (*n* = 4), *P* ≤ 0.1 was used as the threshold for significance to avoid the risk of type II errors, as described in previous FACE analyses of photosynthesis (e.g., Morgan et al., 2004). The biomass data from one AMB plot was identified as an outlier, so the data from this plot were excluded from the analysis.

## Results

### Stimulation of photosynthesis at the end of the season is absent in PH

**Figure 1:**
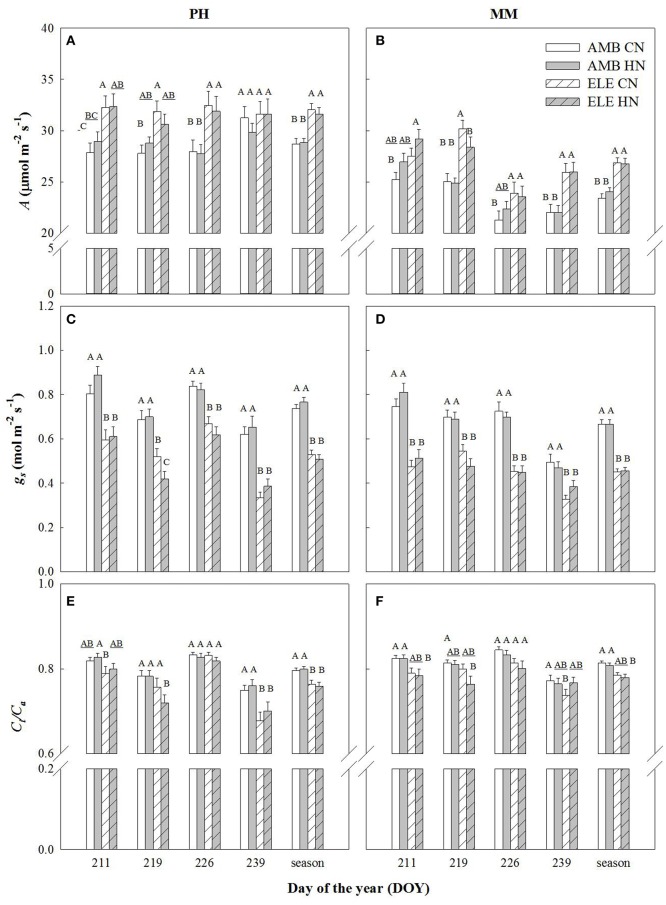
Photosynthetic leaf CO_2_ uptake (*A*; **A,B**), stomatal conductance (*g*_*s*_; **C,D**), and the ratio of internal [CO_2_] to the atmospheric [CO_2_] (*Ci*/*Ca*; **E,F**) in tobacco cultivars Petit Havana (PH) and Mammoth (MM). Bars represent the DOY and season average values for each treatment. AMB CN, ambient [CO_2_] and control N; AMB HN, ambient [CO_2_] and high N; ELE CN, elevated [CO_2_] and control N; ELE HN, elevated [CO_2_] and high N. Error bars are mean ± standard error (SE). Treatments with different letters represent statistically significant differences (*p* < 0.1).

Leaf CO_2_ uptake (*A*) of the most recently expanded leaf, averaged over the growing season, was significantly higher in elevated [CO_2_] (ELE) regardless of cultivar and nitrogen treatment (+11% in PH and +13% in MM). However, this stimulation by ELE was lost in cv. Petit Havanna (PH) by the last measurement date regardless of N treatment (DOY 239), but not in cv. Mammoth (MM) (Figure 1; Table S2). Stomatal conductance (*g*_*s*_) was decreased throughout the season by ELE on average by 32% in both cultivars (Figure 1; Table S2). Mesophyll conductance (*g*_*m*_) decreased 23% under ELE later in the season only in PH (DOY 240; Table S3). Across the season, *C*_*i*_*/C*_*a*_ was modestly but significantly decreased by ELE in PH (−4.5%; Figure 1; Table S2), but not in MM. There was no effect of HN on *A, g*_*s*_, and *C*_*i*_*/C*_*a*_ in either cultivar (Table S2) with the exception of *A* on DOY 211 in MM and of *C*_*i*_*/C*_*a*_ on DOY 211 in PH, which slightly increased with HN by 6.5 and 4% respectively.

### Down-regulation of photosynthesis is strongest in PH under elevated [CO_2_] and control N

**Figure 2:**
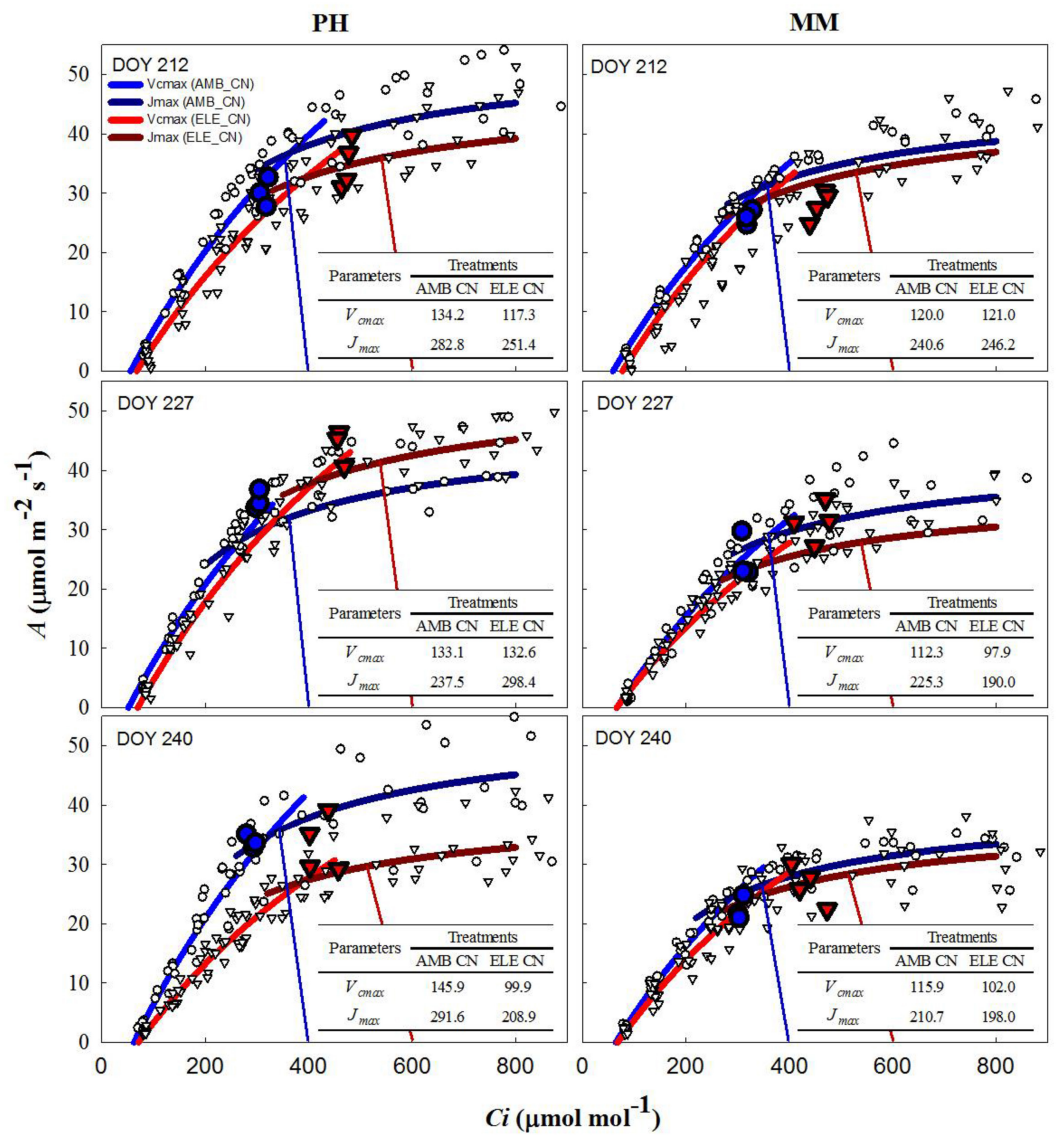
Fitted responses of *A* to *Ci* curves. Inserted tables indicated the daily average *V*_*cmax*_ (μmol m^−2^s^−1^) and *J*_*max*_ (μmol m^−2^s^−1^) for the control N treatments in PH and MM (see also Table S4). Ambient [CO_2_] treatments are represented by blue lines while the elevated [CO_2_] treatments by red lines. The data to which the lines are fit, are shown by white symbols (circles are ambient [CO_2_] and triangles are elevated [CO_2_] treatments). The blue and red vertical lines represent the supply functions (1/-*g*_*s*_) for the ambient and elevated growth [CO_2_], respectively, intercepting the fitted *A/C*_*i*_ at the operating point. The blue (AMB) and red (ELE) large symbols are the treatment means for the midday photosynthesis that were taken the day before of the *A/C*_*i*_ curves. The photosynthetically active radiation (PAR) and air temperature at midday, when these measurements were made, for DOY 211, 226, and 239 were: 1,800, 2,000, and 1,600 μmol m^−2^s^−1^ and 28, 28, and 22.5°C, respectively.

**Figure 3:**
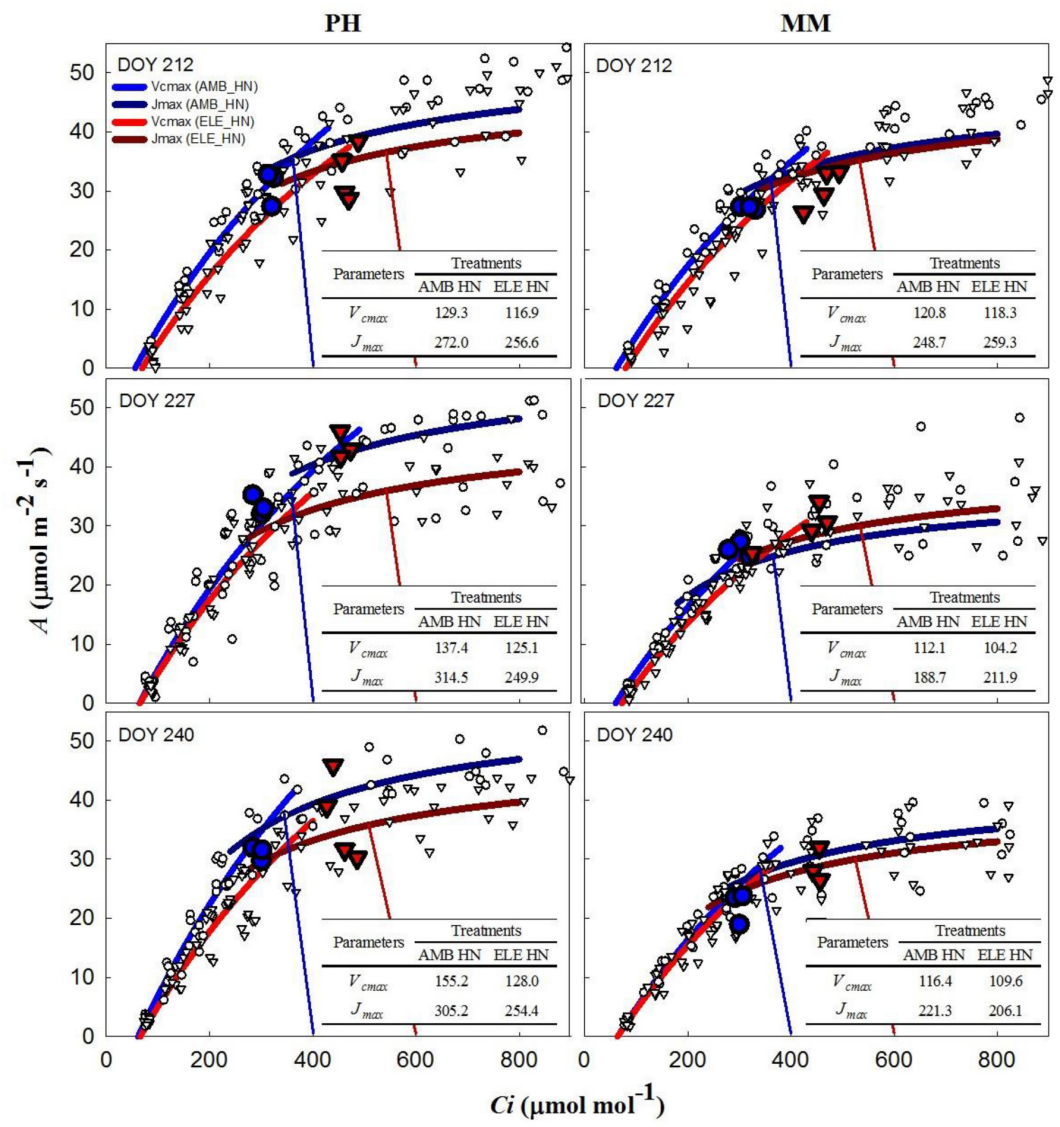
Fitted responses of *A* to *Ci* curves. Inserted tables indicated the daily average *V*_*cmax*_ (μmol m^−2^s^−1^) and *J*_*max*_ (μmol m^−2^s^−1^) for the high N treatments in PH and MM (see also Table S4). Ambient [CO_2_] treatments are represented by blue lines while the elevated [CO_2_] treatments by red lines. The data to which the lines are fit, are shown by white symbols (circles are ambient [CO_2_] and triangles are elevated [CO_2_] treatments). The blue and red vertical lines represent the supply functions (1/-*g*_*s*_) for the ambient and elevated growth [CO_2_], respectively, intercepting the fitted *A/C*_*i*_ at the operating point. The blue (AMB) and red (ELE) large symbols are the treatment means for the midday photosynthesis that were taken the day before of the *A/C*_*i*_ curves. The photosynthetically active radiation (PAR) and air temperature at midday, when these measurements were made, for DOY 211, 226, and 239 were: 1,800, 2,000, and 1,600 μmol m^−2^s^−1^ and 28, 28, and 22.5°C, respectively.

During two of three sets of *A/C*_*i*_ measurements (Table S4), *V*_*cmax*_ and *J*_*max*_ were down-regulated by elevated [CO_2_] in both cultivars. In PH, *V*_*cmax*_ and *J*_*max*_ values obtained from *A/Cc* curves were, on average, 80% higher and 10% lower, respectively, than those obtained from *A/C*_*i*_ curves on DOY 240 (Tables S3, S4). For MM, *V*_*cmax*_ from *A/Cc* curves was ~120% higher and *J*_*max*_ did not change compared to the values calculated from *A/C*_*i*_ curves. However, *V*_*cmax*_ and *J*_*max*_ for either *A/C*_*i*_ and *A/C*_*c*_ curves showed that control nitrogen (CN) treatment had a strong down-regulation in PH, where both the initial Rubisco-limited (*V*_*cmax*_) slope of the response and the upper RuBP regeneration-limited (*J*_*max*_) portion of the response were lower in the ELE plants of this cultivar (Figure 2; Table S4). This down-regulation in PH was so severe on DOY 240, that *A* at the growth [CO_2_], as indicated by the intercept of the supply and demand functions, was lower in ELE than in AMB (Figure 2). HN only partially ameliorated this (Figure 3). In contrast, only a slight down-regulation was evident in MM (Figures 2, 3; Table S3). The operating *C*_*i*_ (i.e., the *C*_*i*_ obtained when the chamber *C*_*a*_ equals the current atmospheric level of 400 μmol mol^−1^) in ELE (see the supply function lines for *g*_*s*_ in Figures 2, 3) is on the *J*_*max*_ limited portion of the response indicating that RubP regeneration was the cause of the observed down-regulation of measured *A*.

### High N fertilization led to a higher leaf N while elevated [CO_2_] increased leaf soluble carbohydrates and starch in both cultivars

Leaf N (g m^−2^) was increased ~10% by the HN treatments in both cultivars. HN had a small but significant effect in leaf C by increasing it 5% in PH and reducing it 6% in MM (Table 1). Leaf C (g m^−2^) was also affected by ELE, which increased by 27% in PH and 31% in MM. Total soluble carbohydrates (TSC; mmol m^−2^) and starch (mmol m^−2^) were significantly higher at noon in ELE compared to AMB, regardless of the cultivar or N treatment. TSC at noon increased by 31% and starch content at noon was almost doubled in both cultivars under ELE (Table 1). By dusk TSC declined by ~50% compared to noon across the treatments while starch remained high in ELE treatments (+46% in PH and +55% in MM). At dawn on DOY 227, TSC and starch were higher in PH in ELE with or without HN compared to control (Table 1).

**Table 1:** On the left side, complete block analysis of variance (ANOVA) with repeated measurements for the season average of specific leaf area (SLA; m^2^ kg^−1^), percentage of leaf nitrogen (leaf N; g m^−2^) and leaf carbon (leaf C; g m^−2^), and total soluble carbohydrates (TSC; mmol m^−2^) and starch (mmol m^−2^) at noon, dusk and dawn.

**CV**	**DOY**	**Parameters**	**Main effects**							**Treatments**							
			**[CO_2_]**	**N**	**[CO_2_] × N**	**Time**	**Time × [CO_2_]**	**Time × N**	**Time × [CO_2_] × N**	**AMB CN**		**AMB HN**		**ELE CN**		**ELE HN**	
PH	Season	SLA	0.003	0.006	ns	<0.0001	ns	ns	ns	22.6 ± 0.6	a	21.2 ± 0.6	b	17.4 ± 0.4	c	16.3 ± 0.4	d
		Leaf N	ns	0.001	ns	0.060	ns	ns	ns	2.13 ± <0.1	b	2.29 ± <0.1	a	1.98 ± <0.1	c	2.26 ± <0.1	ab
		Leaf C	0.006	0.009	ns	<0.0001	ns	0.024	ns	19.20 ± 0.5	d	20.61 ± 0.6	c	24.77 ± 0.7	b	25.60 ± 0.7	a
		TSC noon	<0.001	ns	ns	0.012	ns	ns	ns	18.9 ± 0.9	b	18.2 ± 1.5	b	24.3 ± 1.3	a	24.2 ± 1.0	a
		Starch noon	0.005	ns	ns	<0.0001	ns	ns	0.020	38.5 ± 3.8	b	61.7 ± 9.3	b	110.8 ± 11.7	a	107.7 ± 9.9	a
		TSC dusk	0.053	ns	ns	<0.0001	ns	ns	ns	10.1 ± 0.8	ab	9.8 ± 1.0	b	12.0 ± 0.8	ab	12.7 ± 1.1	a
		Starch dusk	0.79	ns	ns	0.002	0.004	ns	ns	68.0 ± 9.6	c	80.8 ± 13.4	bc	105.1 ± 9.1	ab	112.7 ± 7.9	a
	227	TSC dawn	0.001	0.027	ns	–	–	–	–	9.7 ± 1.4	c	11.8 ± 1.6	bc	15.1 ± 2.0	b	18.5 ± 1.2	a
		starch dawn	0.001	0.048	ns	–	–	–	–	8.3 ± 0.9	b	24.8 ± 8.1	b	65.4 ± 11.1	a	76.7 ± 9.8	a
MM	Season	SLA	0.015	ns	ns	<0.0001	ns	ns	ns	21.5 ± 0.7	a	21.8 ± 0.8	a	15.6 ± 0.5	b	16.8 ± 0.5	b
		Leaf N	ns	0.002	ns	<0.001	ns	ns	ns	1.92 ± <0.1	bc	2.09 ± <0.1	ad	1.95 ± <0.1	bd	2.11 ± <0.1	ac
		Leaf C	0.006	0.086	ns	<0.0001	ns	ns	ns	20.65 ± 0.7	c	20.51 ± 0.7	c	28.24 ± 0.9	a	25.64 ± 0.7	b
		TSC noon	0.024	ns	ns	ns	ns	ns	ns	22.5 ± 1.5	b	23.7 ± 1.4	b	31.0 ± 1.6	a	29.5 ± 1.7	a
		Starch noon	<0.0001	ns	ns	0.001	0.093	ns	ns	56.2 ± 5.6	b	53.8 ± 4.6	b	123.6 ± 9.8	a	117.3 ± 9.0	a
		TSC dusk	ns	ns	ns	0.001	ns	ns	ns	15.3 ± 1.2	a	15.8 ± 1.3	a	17.5 ± 1.1	a	15.6 ± 1.4	a
		Starch dusk	<0.001	ns	0.098	ns	ns	ns	ns	65.8 ± 5.0	c	94.1 ± 10.5	b	125.1 ± 9.2	a	122.2 ± 7.2	a
	227	TSC dawn	ns	ns	ns	–	–	–	–	16.7 ± 2.3	bc	14.1 ± 2.8	c	21.2 ± 1.1	ab	23.3 ± 3.2	a
		Starch dawn	ns	ns	ns	–	–	–	–	51.5 ± 17.5	a	45.0 ± 17.4	a	64.1 ± 13.6	a	72.5 ± 17.4	a

*The ANOVA table also includes the daily analysis of TSC and starch at dawn, no repeated measurements. The statistically significant differences (p < 0.1) and non-statistical significance (ns) are shown in the table. On the right side, values represent day of the year (DOY) and season averages ± standard deviation of the same parameters. Treatments with different letters represent statistically significant differences (p < 0.1)*.

### Effects of CO_2_ and N on SLA, height and total leaf area for PH and MM

Specific leaf area (SLA) decreased ~24% in both cultivars grown in ELE (Table 1). Additionally, SLA decreased slightly (−6%) in PH at HN. Height in PH increased under ELE but decreased at HN, resulting in taller plants at ELE CN than ELE HN (Figure S4 and Table S5). In MM, height was affected by the interaction [CO_2_] × N such that AMB CN plants were taller than AMB HN and ELE CN plants (Figure S4 and Table S5). No significant effects of ELE or HN were found on the number of leaves or branches for either cultivar (Figure S5 and Table S5). However, there was a significant interaction between [CO_2_] and N in the total LA in PH from both harvests (Table S5). Thus, total LA in PH was higher in AMB CN compared to AMB HN on DOY 222, and higher in AMB HN and ELE CN compared to ELE HN on DOY 243 (Figure S5).

### High N reduced biomass at elevated [CO_2_] when compared to control N

**Figure 4:**
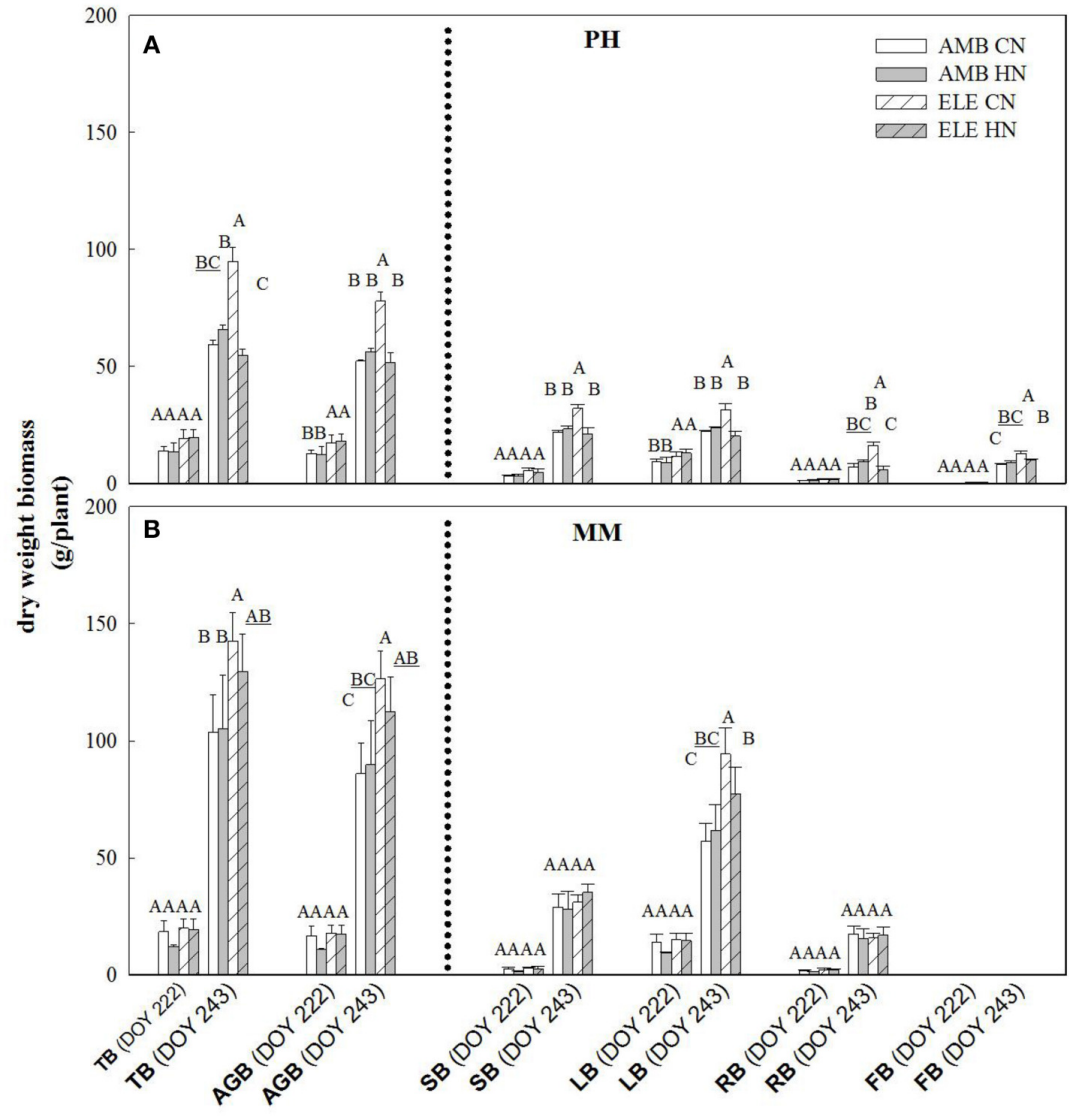
Total (TB), above-ground (AGB), stem (SB), leaf (LB), root (RB) and flower (FB) biomass for PH **(A)** and MM **(B)** tobacco cultivars. The day of the year (DOY) for the two harvests are in parenthesis. Bars represent the average values for each treatment. Cultivars, treatments, error bars and letters above each bar are as in Figure 1.

The yield component of this crop, leaf biomass, was three times greater in MM by comparison to PH at final harvest (DOY 243), regardless of [CO_2_] or N treatment (Figure 4). On this same date, the amount of leaf produced was significantly higher in ELE in both cultivars, but only in CN. Thus, the increase of leaves in PH was 41% and in MM was 65% comparing ELE CN and AMB CN (Figure 4). ELE did not result in significant differences in total biomass in MM by DOY 222 but did by the final harvest on DOY 243 (Figure 4). Curiously, HN had a lesser effect than CN in increasing the total biomass of PH under ELE. Notably, root biomass in ELE HN was less than half of that in CN (−63%; Figure 4).

## Discussion

In this study, two different tobacco cultivars with distinctive growth characteristics and sink potential were grown under fully open-air field conditions at ambient and elevated [CO_2_] with control and high N soil fertilization treatments to address the hypotheses: (1) under high sink capacity conditions represented by the massive leaf growth of cv. Mammoth, down-regulation of photosynthesis will be minimized in elevated [CO_2_] and high N conditions, and (2) as soil N becomes limiting as the growing season progresses, down-regulation of photosynthesis will become more pronounced for both cultivars independently of their sink capacity. Corroborating these hypotheses, the results showed little down-regulation of photosynthesis at elevated [CO_2_] in cv. Mammoth compared to strong down-regulation in cv. Petit Havana (Figures 2, 3). However, HN did not alleviate down-regulation in Mammoth. A progressive down-regulation in *V*_*cmax*_ and *J*_*max*_ was observed in cv. Petit Havana to the extent that by the last sampling date photosynthesis in ELE was less than that in AMB, and was only partially alleviated by HN (Figures 2, 3; Table S4).

The increases in *A* at high [CO_2_] across the season observed in both tobacco cultivars were in the range observed for C_3_ crops in previous FACE experiments (Ainsworth et al., 2002; Long et al., 2004; Ainsworth and Long, 2005; Leakey et al., 2009; Rosenthal et al., 2011). However, the stimulation of *A* at high [CO_2_] was lost in PH by the end of the season (Figure 1) due to strong down-regulation of photosynthetic capacity as reflected in the *A/C*_*i*_ response on DOY 240 (Figures 2, 3). When photosynthesis is limited by the triose phosphate use (TPU-limitation), *V*_*cmax*_ and *J*_*max*_ are usually reduced to match the TPU capacity (Jensen et al., 1987). Thus, it is possible that PH under elevated [CO_2_] was also TPU-limited. However, it is difficult to separate between TPU-limitation and RuBP-limitation since they usually occur under similar conditions (Long and Bernacchi, 2003; Bernacchi et al., 2013).

A decrease in *g*_*s*_ was observed in both cultivars under elevated [CO_2_] (Figure 1 and Table S2), as is common for C_3_ plants grown at elevated [CO_2_] (Drake et al., 1997; Long et al., 2004; Ainsworth and Long, 2005; Ainsworth and Rogers, 2007; Leakey et al., 2009). Despite this decrease in *g*_*s*_, *C*_*i*_*/C*_*a*_ is unaffected by elevation of [CO_2_] in most plants (Long et al., 2004). However, a slight reduction in *C*_*i*_*/C*_*a*_ was seen in PH at elevated [CO_2_] (Figure 1 and Table S2). Otherwise the near constant *C*_*i*_*/C*_*a*_ showed that reduction in *g*_*s*_ does not explain the observed down-regulation of *A* and even in the one case of a significant reduction in *C*_*i*_*/C*_*a*_ it could only account for a very small portion of the reduced *A* (Figures 2, 3). Specific leaf area (SLA) increased in ELE by ~24% indicating thicker leaves and possibly more layers of mesophyll per unit area, since the observed increase in non-structural carbohydrates could not account for this increase (Table 1). A thicker leaf might be expected to have a higher *V*_*cmax*_ and *J*_*max*_, given more resource per unit leaf area. However, these indicators of photosynthetic capacity were not increased in either cultivar and significantly down-regulated in PH despite the increase in SLA. More layers of mesophyll might be expected to cause a decrease in *g*_*m*_ due to the lengthened intercellular gaseous diffusion pathway. On DOY 240, *V*_*cmax*_ calculated from *A/C*_*c*_ curves in PH followed the same trend that *g*_*m*_, decreasing under ELE (Table S3). This pattern was also observed in “apparent” *V*_*cmax*_ (i.e. calculated from *A/C*_*i*_ curves). Thus, the *g*_*m*_ data suggest that part of the observed down-regulation of the “apparent” *V*_*cmax*_ in PH results from a decrease in *g*_*m*_ (Tables S3, S4).

Increased leaf carbohydrates are well document under elevated [CO_2_] growth conditions in many chamber studies (e.g., Stitt, 1991; Stitt and Krapp, 1999) and have been related to the regulation of *V*_*cmax*_ at the level of gene expression (Sheen, 1990; Krapp et al., 1993; Van Oosten and Besford, 1994; Jones et al., 1996; Pego et al., 2000). Our results showed similar amounts of non-structural carbohydrates accumulated in both cultivars under field conditions yet very different levels of down-regulation of “apparent” *V*_*cmax*_ (Figures 2, 3, Table 1). Nevertheless, the findings here do confirm that under open-air conditions of [CO_2_] elevation in an agricultural field down-regulation can be strongly offset in germplasm with a high sink capacity. Therefore, down-regulation of photosynthetic capacity is not inevitable under field conditions where there is no limitation of rooting volume or interference with micro-climate if there is sufficient sink potential and nitrogen supply.

The inability of plants to sustain adequate sinks under elevated [CO_2_] has been linked to nitrogen limitation (Rogers et al., 1996; Rogers and Ainsworth, 2006). Thus, the potential for high N fertilization to sustain *V*_*cmax*_ has been demonstrated, for instance, by Rogers et al. (1998) who observed no acclimation of *V*_*cmax*_ under high N conditions at ambient or elevated [CO_2_] for the perennial ryegrass *Lolium perenne*. Similarly, when N supply was experimentally scaled with the growth of wheat plants under elevated [CO_2_] no down-regulation of *V*_*cmax*_ was observed (Farage et al., 1998). Consistent with these observations HN ameliorated the down-regulation of *V*_*cmax*_ by more than 40% in PH on DOY 240, but N availability did not impact *V*_*cmax*_ in MM. This cultivar difference may result from the fact that PH came into flowering and may experience more N limitation as a consequence of allocation of N to floral structures (Figure 4). It could also result from PH's smaller root system and therefore capacity to access soluble N in the soil, as indicated by root biomass (Figure 4). This interpretation is further supported by the observation that the N in the leaves of PH at the end of the season was significantly lower in ELE CN compared to ELE HN (−23%) agreeing with the notion of N limitation in PH at the end of the season. A relationship between leaf N and the down-regulation of photosynthetic enzymes under FACE elevated [CO_2_] has been proposed previously across a range of species (Ellsworth et al., 2004). This study supports the role of leaf N in the regulation of *V*_*cmax*_ and *J*_*max*_ by high [CO_2_], because leaf N was higher in ELE HN vs. ELE CN through the season in both cultivars (Table 1). In addition, the final harvest increment of total and above-ground biomass in PH under elevated [CO_2_] (59 and 49% in ELE CN vs. AMB CN) was not observed under HN conditions (DOY 243; Figure 4), due perhaps to a very small investment in root biomass (Figure 4).

## Conclusions

The results of this study provide unique evidence that under open-air field conditions, where artificial effects of rooting volume limitation and micrometeorological modification are removed, the commonly reported down-regulation of photosynthesis in elevated [CO_2_] is largely eliminated when sink capacity of germplasm is high and N supply adequate. High sink strength resulting from rapid growth throughout the experiment appears to have prevented down-regulation in tobacco cv. Mammoth whereas the small stature of cv. Petite Havana appears to have resulted in progressive down-regulation. Increased N partially mitigated the down-regulation of photosynthesis in cv. Petit Havana.

## Author contributions

UR, DO, and SL designed the experiment; UR and AD did the field measurements and sample collection; UR performed the leaf carbohydrates and nitrogen analysis and analyzed the data; All auhors discussed the results and contributed to the manuscript writing.

### Conflict of interest statement

The authors declare that the research was conducted in the absence of any commercial or financial relationships that could be construed as a potential conflict of interest.
