# Supplementary material for: The Role of Sink Strength and Nitrogen Availability in the Down-Regulation of Photosynthetic Capacity in Field-Grown Nicotiana tabacum L. at Elevated CO2 Concentration
Source: Front Plant Sci. 2017 Jun 9;8:998. doi: 10.3389/fpls.2017.00998 (PMC5465258; doi:10.3389/fpls.2017.00998)
Supplement: Supplementary file 1 [file Image1.pdf]

Supplemental Material for

The role of sink strength in the down-regulation of photosynthetic capacity in field-grown *Nicotiana tabacum* L. at elevated CO_2 concentration

Ursula M. Ruiz-Vera, Amanda P. De Souza, Stephen P. Long, Donald R. Ort

Correspondence to: d-ort@illinois.edu

Content:

Figures S1 – S5

Tables S1 – S4

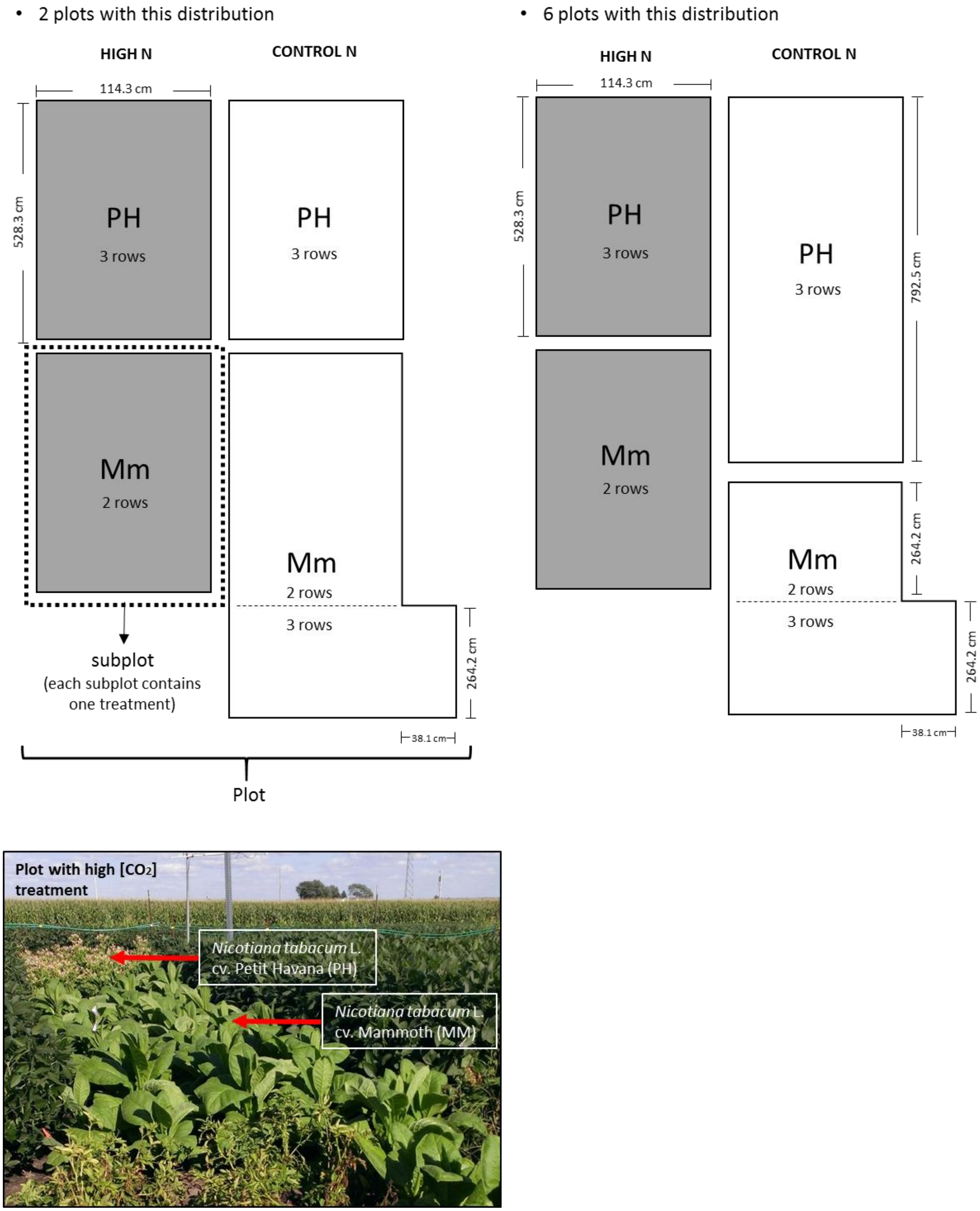

Figure S1. Diagram of the size and of the distribution of the subplots within a CO_2 plot. PH is for tobacco cv. Petit Havana and MM is for Mammoth. Gray subplots represent high N while white subplots are control N fertilization. Photograph of the four subplots within an elevated [CO_2] plot.

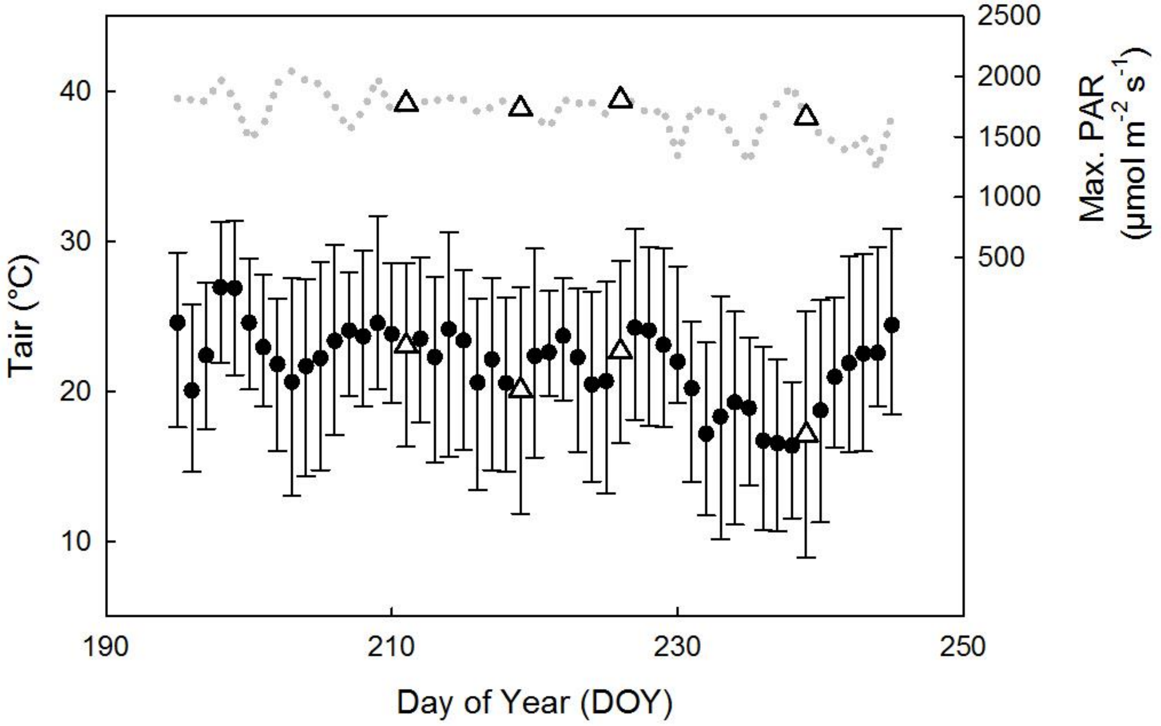

Figure S2. Daily values for the mean air temperature (Tair; black circles) with the maximum and minimum values (top and bottom of the error bars), and maximum photosynthetically active radiation (Max. PAR; dotted-grey line) during the 2015 growing season. Days in which the gas exchange measurements were taken are indicated with white triangles.

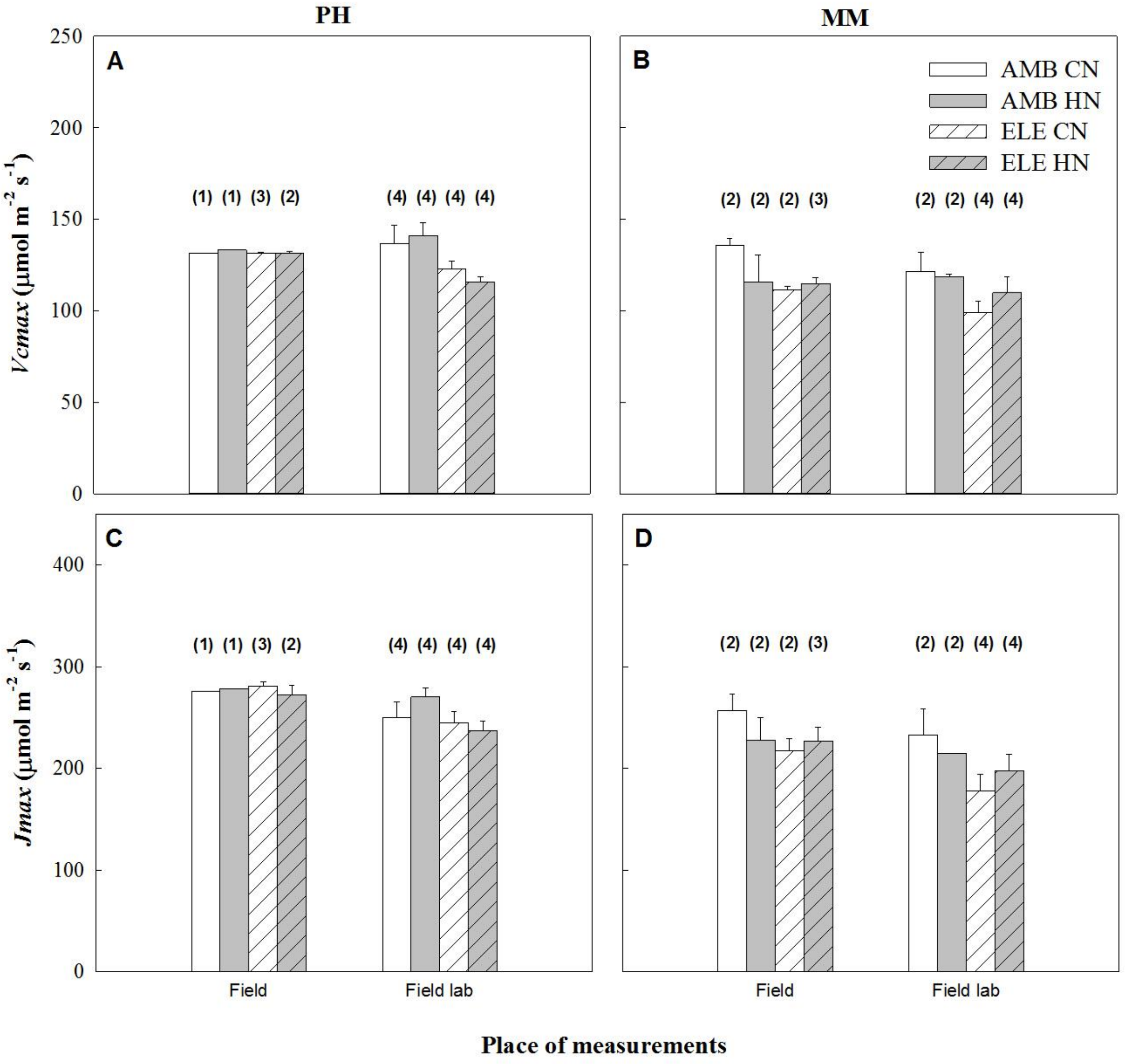

Figure S3. The maximum carboxylation rate by Rubisco (V_{cmax}; A-B), the regeneration of ribulose-1,5-biphosphate controlled by the electron transport rate (J_{max}; C-D) calculated from *A* vs. *Ci* curves taken on DOY 220 in the field (in four plots) and on DOY 221 in the field lab (in the other four plots). Bars represent the daily mean values for each treatment. The numbers above each bar represent the number of values used for the mean. Cultivars, treatments and error bars are as in Figure 1.

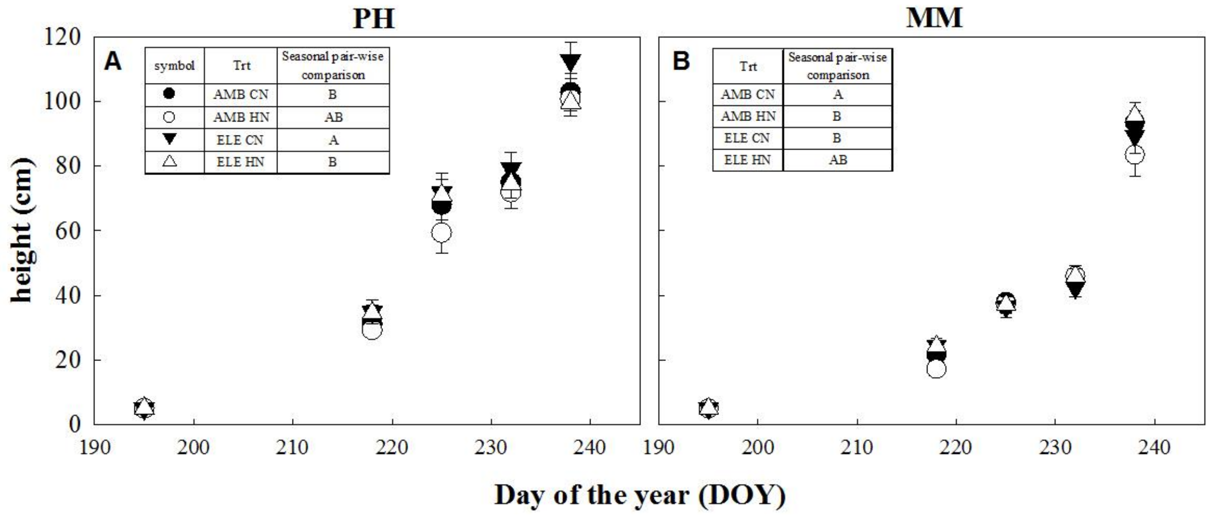

Figure S4. Daily mean for height (A-B) per treatment. Ambient [CO_2] treatments are represented by a circle and elevated [CO_2] treatments by triangles. Black symbols are control N while white symbols are high N fertilization. Cultivars, treatments names and error bars are as in Figure 1.

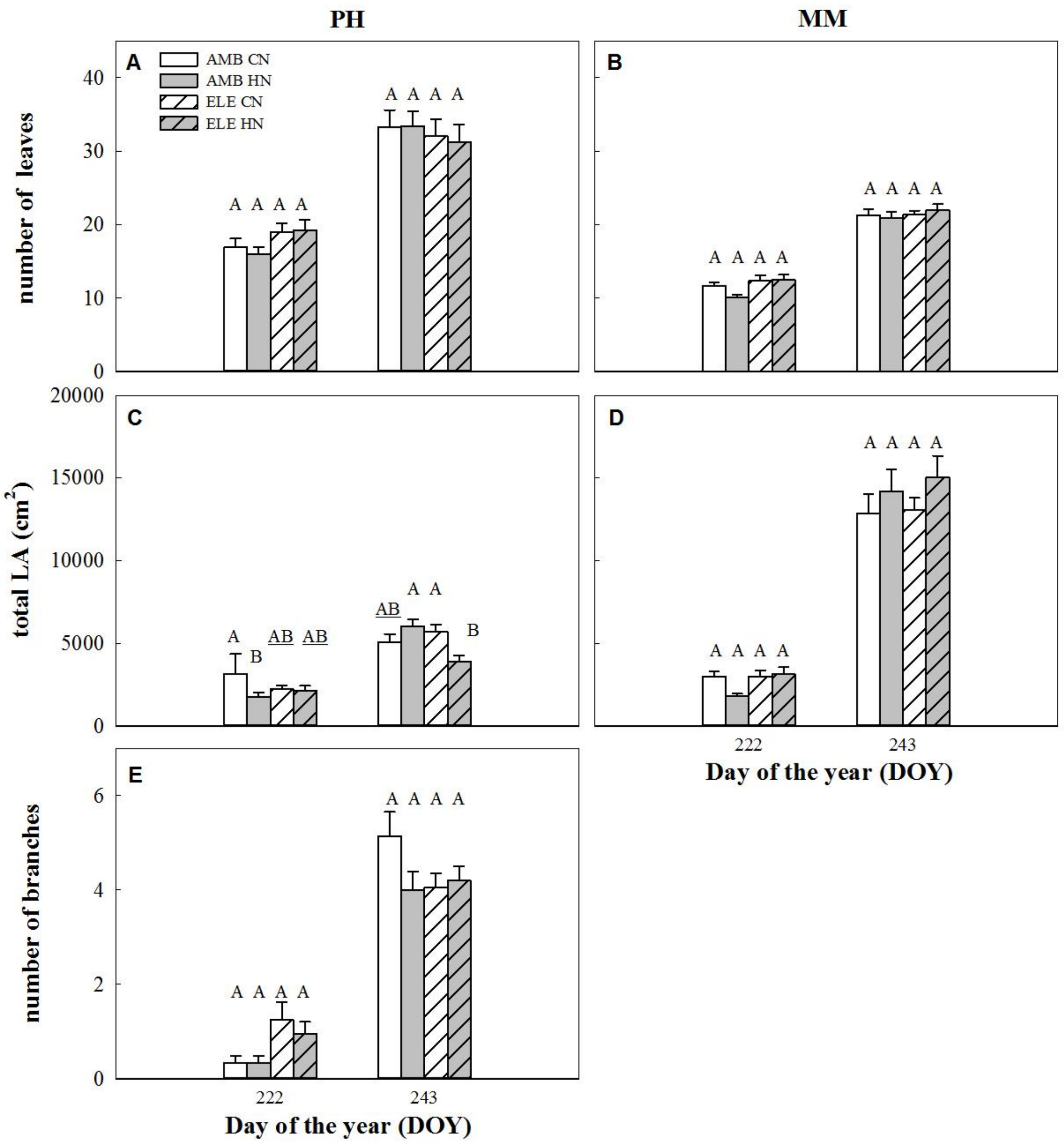

Figure S5. Number of leaves (A-B), total leaf area (total LA; C-D) and number of branches (E) for the 1st and 2nd harvests. Bars represent the daily mean values for each treatment. Cultivars, treatments, error bars and letters above each bar are as in Figure 1.

Table S1. Calendar date and the day of year (DOY) for the measurements on tobacco throughout the season. If the measurements were taken on multiple consecutive days, only the first DOY is indicated. Greenhouse and field planting, germination and soil N fertilization dates are also specified together with the type of developmental growth (vegetative and reproductive) in each tobacco cultivar (PH or MM).

Date	DOY	Measurements	Development	
			PH	MM
15-Jun-15	166	greenhouse planting	vegetative	vegetative
22-Jun-15	173	germination		
13-Jul-15	194	soil N fertilization in the field		
14-Jul-15	195	field planting		
29-Jul-15	210	LAI		
30-Jul-15	211	1st day of gas exchange measurements & leaf sampling		
31-Jul-15 / 1, 2-Aug-15	212	1st set of *A/Ci* curves measurements		
6-Aug-15	218	LAI and height	reproductive	
7-Aug-15	219	2nd day of gas exchange measurements & leaf sampling		
10, 11-Aug-15	222	1st biomass harvest		
13-Aug-15	225	LAI and height		
14-Aug-15	226	3rd day of gas exchange measurements & leaf sampling		
15-Aug-15	227	dawn sampling for leaf carbohydrate analysis		
15, 16-Aug-15	227	2nd set of *A/Ci* curves measurements		
20-Aug-15	232	height		
26-Aug-15	238	LAI and height		
27-Aug-15	239	4th day of gas exchange measurements & leaf sampling		
28, 29-Aug-15	240	3rd set of *A/Ci* curves measurements		
31-Aug-15 / 1, 2-Sep-15	243	2nd biomass harvest		

Table S2. Complete block analysis of variance (ANOVA) with repeated measurements for the DOY data and season average of photosynthetic carbon uptake (*A;* µmolm^{-2}s^{-1}), stomatal conductance ($g_{s;}$ molm^{-2}s^{-1}) and the rate of C_i to the atmospheric [CO_2] (C_i/C_a) from the tobacco cultivars (CV) Petit havana (PH) and Mammoth (MM) which were grown in two different [CO_2] and N conditions. The main effects are: [CO_2], nitrogen (N) and time. The statistically significant differences (p<0.1) and non-statistical significance (ns) are shown in the table.

CV	DOY	Parameters	Main effects						
			[CO_2]	N	[CO_2] x N	time	time x [CO_2]	time x N	time x [CO_2] x N
PH	211	A	0.069	ns	ns	<0.001	ns	ns	ns
		g_s	0.050	ns	ns	<0.0001	ns	ns	ns
		C_i/C_a	ns	0.098	ns	<0.0001	0.008	ns	ns
	219	A	ns	ns	0.099	0.003	ns	ns	ns
		g_s	0.028	ns	0.090	<0.0001	ns	ns	ns
		C_i/C_a	0.057	ns	ns	<0.0001	ns	ns	ns
	226	A	0.009	ns	ns	<0.0001	<0.001	ns	ns
		g_s	<0.001	ns	ns	<0.0001	0.099	ns	ns
		C_i/C_a	ns	ns	ns	<0.0001	0.021	ns	ns
	239	A	ns	ns	ns	<0.001	0.026	ns	ns
		g_s	0.002	ns	ns	0.002	ns	ns	ns
		C_i/C_a	<0.001	ns	ns	<0.0001	0.002	ns	ns
	season	A	0.049	ns	ns	ns	ns	ns	ns
		g_s	<0.0001	ns	ns	<0.0001	ns	ns	ns
		C_i/C_a	<0.0001	ns	ns	<0.0001	0.017	ns	ns
MM	211	A	ns	0.065	ns	0.001	ns	ns	ns
		g_s	0.012	ns	ns	0.009	ns	ns	ns
		C_i/C_a	0.084	ns	ns	<0.0001	0.068	ns	ns
	219	A	0.103	ns	ns	0.030	ns	ns	ns
		g_s	<0.0001	ns	ns	<0.0001	ns	ns	ns
		C_i/C_a	ns	ns	ns	<0.001	ns	ns	ns
	226	A	0.097	ns	ns	<0.0001	0.016	ns	ns
		g_s	<0.0001	ns	ns	0.036	ns	ns	ns
		C_i/C_a	ns	ns	ns	<0.0001	ns	ns	ns
	239	A	0.001	ns	ns	0.001	ns	ns	ns
		g_s	<0.001	ns	ns	0.010	ns	ns	ns
		C_i/C_a	ns	ns	ns	<0.0001	ns	ns	ns
	season	A	0.032	ns	ns	<0.0001	ns	ns	ns
		g_s	<0.0001	ns	ns	<0.0001	0.006	ns	ns
		C_i/C_a	ns	ns	ns	<0.001	ns	ns	ns

Table S3. On the left side, complete block analysis of variance (ANOVA) for the DOY 240 of mesophyll conductance (g_m; mol m^2 s^{-1}), the maximum carboxylation rate by Rubisco (V_{cmax}; µmol$m^{-2}s^{-1}$) and the regeneration of ribulose-1,5-biphosphate controlled by the electron transport rate (J_{max}; µmol$m^{-2}s^{-1}$) from *A* vs. *Cc* curves. The tobacco cultivars (CV) Petit havana (PH) and Mammoth (MM) were grown under two [CO_2] and N conditions. The statistically significant differences (p<0.1) and non-statistical significance (ns) are shown in the table. On the right side, values represent the averages ± standard deviation of the same parameters. Treatments with different letters represent statistically significant differences (p<0.1).

CV	DOY	Parameters from *A/Cc*	Main effects			Treatments			
			[CO_2]	N	[CO_2] x N	AMB CN	AMB HN	ELE CN	ELE HN
PH	240	g_m	0.028	ns	ns	0.127 ± 0.01 ab	0.141 ± 0.01 a	0.091 ± 0.01 c	0.115 ± 0.01 bc
		V_{cmax}	0.018	0.054	0.073	281.0 ± 14.7 a	283.4 ± 14.2 a	181.2 ± 15.3 c	227.3 ± 20.2 b
		J_{max}	0.002	0.077	ns	252.4 ± 13.5 a	255.0 ± 7.6 a	200.4 ± 9.9 c	228.9 ± 12.4 b
MM	240	g_m	ns	ns	ns	0.082 ± 0.01 a	0.090 ± 0.01 a	0.082 ± 0.01 a	0.091 ± 0.01 a
		V_{cmax}	0.070	ns	ns	269.0 ± 21.3 ab	274.3 ± 21.1 a	214.3 ± 15.6 b	236.0 ± 25.0 ab
		J_{max}	ns	ns	ns	200.9 ± 8.5 a	209.3 ± 11.8 a	202.2 ± 6.3 a	197.1 ± 9.4 a

Table S4. On the left side, complete block analysis of variance (ANOVA) for the DOY data and for the season average (with repeated measurements) of the maximum carboxylation rate by Rubisco (V_{cmax}; µmolm^{-2}s^{-1}) and the regeneration of ribulose-1,5-biphosphate controlled by the electron transport rate (J_{max}; µmolm^{-2}s^{-1}) from *A* vs. *Ci* curves taken three times during the growing season in tobacco. The tobacco cultivars (CV) Petit havana (PH) and Mammoth (MM) were grown under two [CO_2] and N conditions. The statistically significant differences (p<0.1) and non-statistical significance (ns) are shown in the table. On the right side, values represent DOY and season averages ± standard deviation of the same parameters. Treatments with different letters represent statistically significant differences (p<0.1).

CV	DOY	Parameters from *A/Ci*	Main effects							Treatments							
			[CO_2]	N	[CO_2] x N	time	time x [CO_2]	time x N	time x [CO_2] x N	AMB CN		AMB HN		ELE CN		ELE HN	
PH	212	V_{cmax}	<0.001	ns	ns	—	—	—	—	134.2 ± 8.0	a	129.3 ± 5.9	a	117.3 ± 7.5	b	116.9 ± 7.6	b
		J_{max}	0.001	ns	ns	—	—	—	—	282.8 ± 18.5	a	272.0 ± 17.1	a	251.4 ± 19.5	b	256.6 ± 18.5	b
	227	V_{cmax}	ns	ns	ns	—	—	—	—	133.1 ± 3.7	a	137.4 ± 9.0	a	132.6 ± 5.3	a	125.1 ± 6.5	a
		J_{max}	ns	ns	0.034	—	—	—	—	237.5 ± 13.3	b	314.5 ± 11.2	a	298.4 ± 22.8	ab	249.9 ± 15.9	b
	240	V_{cmax}	0.046	0.005	0.061	—	—	—	—	145.9 ± 10.4	ab	155.2 ± 8.7	a	99.9 ± 4.5	c	128.0 ± 8.0	b
		J_{max}	0.043	ns	ns	—	—	—	—	291.6 ± 29.2	a	305.2 ± 13.2	a	208.9 ± 11.3	b	254.4 ± 12.7	a
	season	V_{cmax}	<0.001	ns	ns	ns	0.026	ns	ns	137.7 ± 7.4	a	140.6 ± 7.8	a	116.6 ± 5.7	b	123.3 ± 7.4	b
		J_{max}	0.013	ns	ns	ns	ns	ns	0.058	270.7 ± 20.4	ab	297.2 ± 13.9	a	252.9 ± 17.9	b	253.7 ± 15.7	b
MM	212	V_{cmax}	ns	ns	ns	—	—	—	—	120.0 ± 0.2	a	120.8 ± 3.7	a	121.0 ± 2.2	a	118.3 ± 1.6	a
		J_{max}	ns	ns	ns	—	—	—	—	240.6 ± 6.5	a	248.7 ± 5.5	a	246.2 ± 15.6	a	259.3 ± 6.3	a
	227	V_{cmax}	0.067	ns	ns	—	—	—	—	112.3 ± 6.6	a	112.1 ± 7.7	a	97.9 ± 3.5	b	104.2 ± 4.3	ab
		J_{max}	ns	ns	0.074	—	—	—	—	225.3 ± 13.1	a	188.7 ± 17.0	ab	190.0 ± 6.6	b	211.9 ± 10.5	ab
	240	V_{cmax}	0.051	ns	ns	—	—	—	—	115.9 ± 2.7	a	116.4 ± 5.5	a	102.0 ± 6.0	b	109.6 ± 5.9	ab
		J_{max}	ns	ns	ns	—	—	—	—	210.7 ± 8.6	ab	221.3 ± 15.0	a	198.0 ± 11.6	b	206.1 ± 11.0	ab
	season	V_{cmax}	0.007	ns	0.002	ns	ns	ns	ns	116.1 ± 3.2	a	116.4 ± 5.6	a	107.0 ± 3.9	b	110.7 ± 3.9	ab
		J_{max}	ns	ns	<0.0001	ns	ns	ns	ns	225.5 ± 9.4	a	219.6 ± 12.5	ab	211.4 ± 11.3	b	225.8 ± 9.3	a

Table S5. Complete block analysis of variance (ANOVA) with repeated measurements for height (cm), number of leaves, total leaf area (total_LA; cm^2), number of branches (per plant), total, above-ground, stem, leaf, root and flowers biomass (g per plant) from tobacco PH and MM. The statistically significant differences (p<0.1) and non-statistical significance (ns) are shown in the table.

CV	Parameters	Main effects						
		$[CO_2]$	N	$[CO_2]$ x N	time	time x $[CO_2]$	time x N	time x $[CO_2]$ x N
PH	height	0.024	0.023	ns	<0.0001	ns	ns	ns
	number of leaves	ns	ns	ns	<0.0001	ns	ns	ns
	total LA	ns	ns	ns	<.0001	ns	ns	0.012
	number of branches	ns	ns	ns	<00001	0.095	ns	ns
	total biomass	0.002	0.004	<0.001	<0.0001	ns	0.003	<0.001
	above-ground biomass	0.002	0.025	0.004	<0.0001	ns	0.020	0.002
	stem biomass	0.019	0.044	0.013	<0.0001	ns	0.079	0.020
	leaf biomass	0.031	ns	0.045	<0.0001	ns	0.049	0.015
	roots biomass	0.056	0.014	0.001	<0.0001	ns	0.017	0.001
	flowers biomass	0.001	ns	0.021	<0.0001	0.002	ns	0.020
MM	height	ns	ns	0.020	<0.0001	ns	ns	ns
	number of leaves	ns	ns	ns	<0.0001	ns	ns	ns
	total LA	ns	ns	ns	<0.0001	ns	ns	ns
	total biomass	ns	ns	ns	<0.0001	0.081	ns	ns
	above-ground biomass	ns	ns	ns	<0.0001	0.043	ns	ns
	stem biomass	ns	ns	ns	<0.0001	ns	ns	ns
	leaf biomass	0.102	ns	ns	<0.0001	0.026	ns	ns
	roots biomass	ns	ns	ns	<0.0001	ns	ns	ns
